# On the Treatment of After-Pains

**Published:** 1847-05

**Authors:** Edward Rigby

**Affiliations:** Senior Physician to the General Lying-in Hospital, &c., &c.


*On the Treatment of After-Pains.* By Edward Rigby, M. D., Senior Physician to the General Lying-in Hospital, &c., &c.—We find these pains after most labors, but they vary much, both in duration and severity. They are always most severe in women who have borne many children, and in some multiparæ they continue from 24 hours to three days. These pains, in common with those of labor, depend on contraction of the uterus; and these contractions are kept up after labor, by the presence of the lochia, or of coagula of blood, or of shreds of membrane in the cavity of the uterus. The after-pains, therefore, you will easily understand, are essential to the duty of this organ. After-pains are not usually felt in primiparæ. In multiparæ, when the woman is quite healthy, they seldom continue very long. When very severe, and of very long continuance, they may be considered as indicating the presence of inflammation, or, at all events, of a state bordering on inflammation. The pain no longer comes and goes in distinct intermissions, and it gradually becomes constant; the uterus, too, becomes tender on pressure, and the other symptoms of inflammation are established, but we shall have to consider this subject hereafter, when speaking of puerperal fever. It must, however, be borne in mind, that long-continued and severe after-pains may form an insidious transition to a state of inflammation.

The treatment pursued during the last few days of pregnancy has great influence in modifying the after-pains. If the woman be allowed to go, even up to the period of her labor, with her bowels confined and loaded with unhealthy fæces, and with the secretions of the primæ viæ disordered, the uterus seldom contracts well during labor, and, under these circumstances, after-pains are sometimes very severe. The too rapid expulsion of the placenta, and the hasty conclusion of the last stage of labor, so that the uterus does not contract fully and firmly, are also causes of the contractions which cause these after-pains; and, in these cases, the after-pains are useful in expelling the clots and lochia, which, if allowed to remain, would become putrid, and thus prove a source of irritation, and, perhaps, give rise to puerperal fever. When the practitioner neglects to apply the child to the breast as soon as labor is concluded, the uterus does not contract fully, as before mentioned; this, therefore, is another reason for early suckling. In speaking of the application of the child to the breast, it was omitted to mention the fact of nurses having a saying, that "the child brings after-pains." This occurs to a great extent, when the infant is not applied to the breast until the third day, and is, therefore another reason, if more were required, for its early application.

Although after-pains are commonly, no doubt, excited by the presence of coagula in the cavity of the uterus, still they may sometimes occur when no such coagula are to be found. On this subject Dr. Burton, of York, who published in 1751, has given some curious observations, which explain one cause of after-pains very satisfactorily. Dr. Burton says, "upon the expulsion of the child and the placenta, the orifices of the uterine sinuses must contract, and thus retain the grumous blood which is in them; hence the use and benefit of these after-pains, which by stimulating and compressing the vessels and muscular fibres, make them exert their force to squeeze out this grumous blood, which otherwise might remain there, and occasion inflammation, suppuration, &c., from all which, we find that these after-pains are necessary towards removing or preventing an inflammation of the womb; therefore, we must not be too forward in giving strong opiates, and other internal medicines, which may take them off whilst this grumous blood is lodged within those sinuses. I doubt not," continues Dr. Burton, "that those patients who die from the eighth to the fourteenth day, whose uterus has been inflamed with the symptoms above mentioned, have been injured by the too free use of opiates." The discharge, in these cases, contains little vermiform shreds, and these shreds do not consist of portions of membrane which have been left behind, but they are the casts or moulds, as it were of the cavities of the bloodvessels, and are composed of their contents coagulated. Dr. Burton mentions a case to which he was called, in consequence of severe after-pains coming on some time after labor; in this case, having introduced his hand with some difficulty, he perceived several small membranous strings, as he then thought them, adhering to the uterus; but, he says, "I was soon undeceived, for, upon expanding my fingers, by which I stretched the womb a little, several of these came into my hand, which I drew out, and found what I had imagined to be membranes to be only oblong grumous blood, resembling fibres, like those that adhere to a spatula after stirring arterial blood in a basin for some time. I introduced my hand a second time," he continues, "and made the experiment again, but found none of these little clots within the cavity of the womb; yet, upon expanding my hand, several came out of the orifices again, which I could plainly perceive, and, after keeping my hand there a little while, I brought away all that were in the cavity of the uterus, and the patient's complaints immediately abated, and she recovered well from that moment." These little vermiform fibres are, therefore, the casts of the bloodvessels, the contents of which had coagulated, having been retained by the contraction of their orifices. By being careful not to hasten the last stage of labor, so as to give the uterus time to contract slowly, the uterine sinuses expel their contents, and the annoyance of after-pains is, in many cases altogether prevented.

It was formerly the practice always to administer a large dose of laudanum, to check these after-pains; and a most pernicious practice it was, but which happily has diminished considerably of late years. As much as forty minims of tincture of opium were administered as a rule in all cases by some accoucheurs. It is hardly necessary to point out the falsity of the reasoning on which this practice was founded. Opiates should never be given for the purpose of checking after-pains, unless they are very severe, or in some peculiar cases which will be hereafter considered. Should the patient, however, have been accustomed to an after-pain draught, as it is called, and seem to expect it, a little tincture or extract of hyoscyamus or of lettuce may be given her. A mild sedative may, however, in many cases be useful after labor, as when the system is in a state of irritability and restlessness, consequent on the fatigue and irritation of a lengthened period of suffering; but in these cases a small dose of Dover's powder, or of the sedative solution of opium, will always prove sufficient. When after-pains continue very severe for some time after labor, the best plan of treatment will be to give the customary dose of castor oil a little earlier than usual; this will generally relieve the pains, on bringing a quantity of fecal matter. When the pains continue very severe, in spite of this treatment, the presence of inflammatory action may be suspected, and the treatment must be accordingly.—*Medical Times.—Ibid.*

